# Individualizing Treatment Approaches for Epileptic Patients with Glucose Transporter Type1 (GLUT-1) Deficiency

**DOI:** 10.3390/ijms19010122

**Published:** 2018-01-05

**Authors:** Armond Daci, Adnan Bozalija, Fisnik Jashari, Shaip Krasniqi

**Affiliations:** 1 Department of Pharmacy, Faculty of Medicine, University of Prishtina, Prishtina 10000, Kosovo; armond.daci@uni-pr.edu (A.D.); adnan.bozalija@uni-pr.edu (A.B.); 2 Institute of Pharmacology and Toxicology, Faculty of Medicine, University of Prishtina, Prishtina 10000, Kosovo; 3 Department of Neurology, University Clinical Center of Kosovo, Prishtina 10000, Kosovo; fisnik.jashari@medicin.umu.se

**Keywords:** glucose transporter type-1 deficiency, SLC2A1, epilepsy, pharmacogenomic, diet

## Abstract

Monogenic and polygenic mutations are important contributors in patients suffering from epilepsy, including metabolic epilepsies which are inborn errors of metabolism with a good respond to specific dietetic treatments. Heterozygous variation in solute carrier family 2, facilitated glucose transporter member 1 (SLC2A1) and mutations of the GLUT1/SLC2A2 gene results in the failure of glucose transport, which is related with a glucose type-1 transporter (GLUT1) deficiency syndrome (GLUT1DS). GLUT1 deficiency syndrome is a treatable disorder of glucose transport into the brain caused by a variety of mutations in the SLC2A1 gene which are the cause of different neurological disorders also with different types of epilepsy and related clinical phenotypes. Since patients continue to experience seizures due to a pharmacoresistance, an early clinical diagnosis associated with specific genetic testing in SLC2A1 pathogenic variants in clinical phenotypes could predict pure drug response and might improve safety and efficacy of treatment with the initiation of an alternative energy source including ketogenic or analog diets in such patients providing individualized strategy approaches.

## 1. Introduction

Epilepsy is a disease that cannot be described by only a single condition, but rather represents a family of diverse disorders having in common an abnormally increased predisposition to seizures, which occur due to abnormal, excessive, or synchronous brain neuronal activity [1]. It affects 65 million people worldwide and it is most common chronic and serious neurological disease [2]. Although different epileptic syndrome pathophysiologies can be identified, their appearance shares common characteristics such as increased and persistent neuron excitability and synchronicity, as well as misbalance between inhibitory and excitatory neurons in the brain [3].

Moreover, genetic background contributes in 40% of patients suffering from epilepsy; this includes monogenic and polygenic mutations. Based on this background, genetic basis is the second approach to be detected further with promising indications [4]. Despite molecular mechanisms of actions not being completely understood, there are a number of classes of therapeutics that are currently used in epilepsy [5].

Metabolic epilepsies are inborn errors of metabolism which respond to specific treatments based on a diet or supplementation alone or in combination with conventional treatment [6].

The ketogenic diet (KD) is a high-fat, low protein, and low-carbohydrate diet (4 g of fat to every 1 g of carbohydrate and protein combined (4:1 ratio) and classic (3:1 ratio). It reduces consumption of glucose from the brain and provides ketone bodies as alternative sources, which have a mechanism of action through GABA synthesis and reception, resulting in a neuroprotective effect and antiepileptogenesis.

The KD is used in drug-resistant epilepsy, young epileptic patients and infants, epilepsy syndromes, absence seizures, rett syndrome, tuberous sclerosis, refractory and secondary generalized epilepsy. Moreover, the other diet type including the modified atkins diet (MAD) contains unlimited proteins and provides carbohydrates amounts in lower amounts for children (10 g/day) compared to adults (15 g/day) with a 1:1 fat-to-nonfat ratio and is less restrictive alternative compared to the classic KD in terms of discipline and maintaining the compliance. However, using the MAD in the GLUT1 DS patients were shown to have comparable efficacy with KD with a lower ketosis, improved taste, easier daily management, and better selection of dietary foods [7,8,9]. Differences exist also in the indications of this diet corresponding to clinical phenotypes and age groups as follows: the classic ketogenic diet 4:1 carbohydrate is indicated in severe phenotypes in infants; the classic ketogenic diet 3:1 carbohydrate is indicated more in mild phenotypes in infants, both phenotypes in pre-school age, school age, adolescent, adults; and MAD is indicated in mild phenotypes in school age, adolescents, and adults [9,10].

Recently, there have been significant improvements in genetic testing, including gene variations that could predict drug response and also improve safety and efficacy of antiepileptic drugs. There are different genes that have been identified can affect drug response by influencing pharmacokinetics—such as transporters and its systems, metabolism, and elimination—drug targets which are involved in the brain excitability. This also provides molecules and enzyme expressions in the pathogenesis of adverse reactions [11].

Since patients continue to experience seizures due to pharmacoresistance [12], there are different efforts and few examples when pharmacogenomic testing can be recommended in clinical practice, which provides better drug response or may predict the effective dose. However, currently, there is no identified biomarker with broadly applicable importance.

Recently, there is a tendency to focus on the personalization of therapy in the development of more applicable biomarkers and also an application of treatments or drugs which therapeutically could reverse molecular defects in epilepsy causes from genetic defects. It has also been shown that some antiepileptic treatments exacerbate seizures. Because of this, selection of appropriate antiepileptic therapy plays an important role in the personalization of therapy in responding to epilepsy genetic defects [13,14].

## 2. Glut1 Deficiency Syndrome Treatment Individualizing Approaches

Individualized treatment is based on the individual variability of genes by identification of underlying genetic etiology. Several strategies that could achieve this, including detection of causative genetic alterations, determination of functional alterations of the physiological system that are caused by the genetic mutation with an impact on reversing or inhibiting that functional alteration, and improvement of epilepsy treatment by targeting the biological mechanisms responsible for epilepsy in each individual [15].

Heterozygous variation in solute carrier family 2, facilitated glucose transporter member 1 (SLC2A1) and mutations of the GLUT1/SLC2A2 gene results in the failure of glucose transport, which is related with a glucose type-1 transporter (GLUT1) deficiency syndrome (GLUT1DS); this affects the transport of glucose across the blood–brain barrier and its availability in the brain [16,17]. This mutation is a result of haploinsufficiency of the SLC2A gene and a reduced expression of its translated products, including GLUT1 [18]. The affected patients display low glucose levels in the cerebrospinal fluid (CSF) and hypoglycorrhachia [19]. This is more common in young age and is manifested with infantile seizures that are resistant to conventional antiepileptic drugs, non-convulsive status epilepticus predominantly in fasting state, myoclonic-astatic epilepsy, paroxysmal exercise-induced dyskinesia, severe psychomotor retardation, dystonic features, ataxia, developmental delay, acquired microcephaly, hypotonia, spasticity and movement disorder, and also wide phenotypic pleiotropy, including intellectual disability, movement disorder, and drug-resistant epilepsy. There are rare conditions in generalized genetic epilepsies in the mutations of SLC2A1 which are found in early-onset absence seizures (EOAE) before the age of four and rarely classical juvenile absence epilepsy [20,21,22].

This can manifest with a variety of phenotypes including early-onset epileptic encephalopathy and early childhood-onset refractory absence epilepsy [23].

Although the generalized forms of epilepsy were not related to SLC2A1 mutations in some studies, there are reports that SLC2A1 mutations in combination with associated movement disorders, motor, and/or speech difficulties may be reflected in generalized epilepsy [24,25]. Similarly, in an additional study, a 2.1% SCL2A1 mutation had generalized epilepsy and movement disorders, abnormal movements, and family history of seizures [26].

Despite the tendency to include detection of other transporters with polymerase chain reaction and Sanger sequencing, GLUT1 remains the only metabolism transporter associated with generalized epilepsy. However, certain types of pathogenic variants may exist in these genes, which could not be detected with Sanger sequencing [27].

When this metabolic error is corrected, the brain functions may be almost completely restored, which is makes it essential to diagnose early and start with the ketogenic diet as soon as possible [28].

Epilepsy-related with GLUT-1 deficiency is associated with a poor response to a standard antiepileptic medication therapy [15]. In the therapeutically difficult patients with SLC2A1 mutations, the benefit from the ketogenic diet should also be considered in genetic generalized epilepsies.

In early infantile epileptic encephalopathies (EIEE) and severe epilepsies of infancy, SLCA1 mutations are rare and the identifications for these patients for specific therapy including ketogenic diet is an important strategy to be implemented [20]. The milder forms of epilepsy due to GLUT1 deficiency may respond to standard antiepileptic drugs even though there are currently no approved medicines for Glut1 DS; however, when unresponsive to medications or seizures that are typically refractory to medical treatments, the general gold standard treatment of these patients includes the ketogenic diet, which contributes to treating the symptoms of neuroglycopenia through bypasses in the defective glucose transport and alternative energy supply to the brain. Based on this, an early intervention is crucial for seizure control [14,29].

Concerns have been raised as the ketogenic diet induces an increase in cholesterol levels, increases the risk of metabolic syndrome, transiently increases lipids in children, and increases cardiovascular risk factors in adults. Direct replenishment of energy substrates feeding into the TCA-cycle represents an alternative mechanism to decrease seizure activity. The rationale of treating patients with GLUT1-DS is that triheptanoin (anaplerotic substrate) with its metabolized products including heptanoate and C4 and C5 carbon compound ketone bodies easily cross the blood–brain barrier via monocarboxylate transporters and may enhance the effect of the regular ketones as an alternative fuel for the brain. Due to this, there are positive preclinical studies performed in epilepsy-related GLUT1-DS [30] with ongoing clinical trials [31]. A recent clinical report has shown that triheptanoin resulted in a 90% clinically improvement in GLUT-DS patients with nonepileptic paroxysmal manifestations previously objected to or intolerant in KD [32]. Similarly, another open-label study conducted in GLUT1-DS patients showed a reduction in spike waves on electroencephalogram after 90 min of treatment with triheptanoin [33]. Taking this into consideration, anaplerosis allows better carbohydrate intake and glucose levels relative to KD without exacerbation of the deficiency of glucose transport into the brain which further indicates promising therapeutic strategy in the future, even though there are larger controlled studies required to better establish a therapeutic approach in patients who object to KD or cannot comply with its constrictions [34,35].

**Figure 1:**
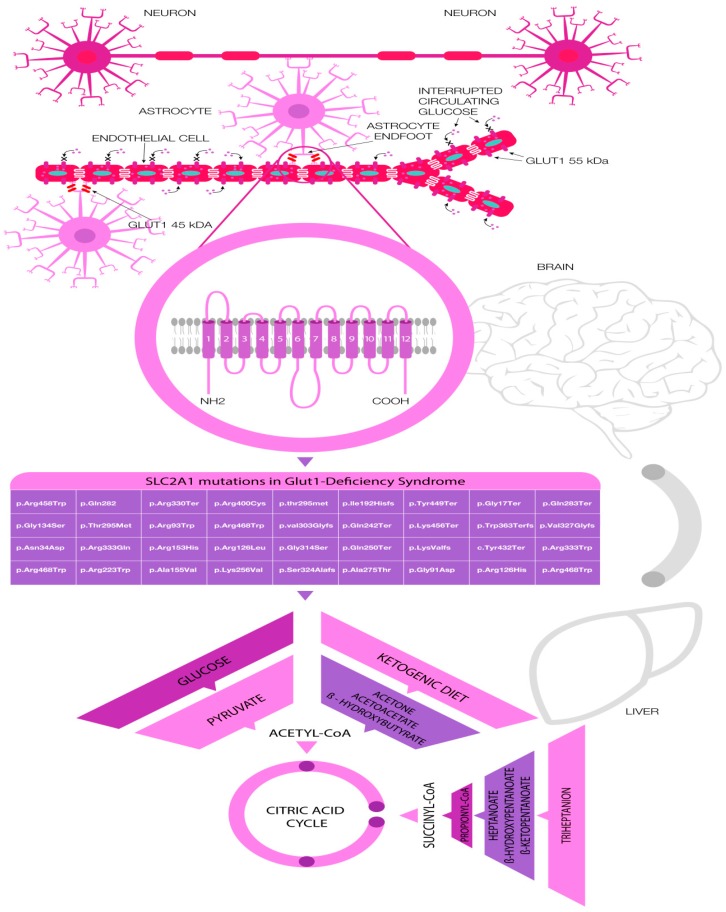
Disorder of glucose transport (GLUT1-DS) across the blood–brain barrier and its availability in the brain (astrocytes and neurons respectively) caused by the variety of mutations in the SLC2A1, genetic testing of pathogenic variants, and therapeutic approaches with alternative energy sources.

However, the more flexible diets, including modified Atkins diet and carbonic anhydrase inhibitors such as acetazolamide or zonisamide can be effective in milder cases by reducing the seizure control, improvement of movement disorders, and dyskinesia [36,37]. These alternative energy sources are depicted in Figure 1.

Careful consideration must be given for adjunctive treatment in children on the ketogenic diet and adjunctive antiepileptic drugs—including phenobarbital, valproic acid (through inhibiting Glut1 transport), valproic acid (inhibition of beta-oxidation of fatty acids), acetazolamide, topiramate, and zonisamide (inhibition of carbonic anhydrase which potentiate metabolic acidosis and kidney stones)—and patients affected with Glut1-DS are advised to avoid barbiturates (especially in children with infantile-onset-seizures), caffeine, and valproic acid due to unpredicted clinical consequences of its use [38,39,40]. Lately, the gene therapy in mouse models produced exogenous GLUT1 in neural cells and also improved CSF glucose levels suggesting that this will be an effective human therapy in the future [41]. However, the summarizing of all the possible treatment options for GLUT1 deficiency syndrome related to epilepsy is shown in Table 1.

## 3. Genetic Testing in Glut1-DS

Diagnosis may go undetected if comprehensive laboratory investigations are not undertaken, including urine and CSF analysis, MR spectroscopy or detection of inborn errors of metabolism in the early onset refractory epilepsies with unclear etiology, especially with epilepsy phenotype including the seizures for suggestive of Glut-1 deficiency syndrome which also requires additional metabolic studies to provide a diagnosis rapidly that can impact the management [19].

Genetic testing has a pre-test probability of 1% for idiopathic generalized epilepsy (IGE) and 10% for early-onset absence epilepsy [15,20]. Glut1 defects are more common in IGE, featuring absence epilepsies with onset from early childhood to adult life [42].

Despite the clinical diagnosis, the genetic testing approaches in the SLC2A1 gene mutations are detected in 70–80% of patients with GLUT1DS and should be reserved for those patients with borderline clinical and investigational findings prior to initiation of diet [14].

In a study performed in Japanese patients with hypoglycorrhachia and typical clinical symptoms of Glut1-DS, there were 33% patients without mutations in exons and exon–intron boundaries of the SLC2A1 gene detected by direct sequencing, which potentially could be underdiagnosed in the promoter sequences and/or sequences deep with introns [43].

If the patients are negative in these mutations and there is still strong clinical suspicion present, they should also be treated with diet.

The GLUT-1 deficiency challenge also has a possible genetic diagnosis in epilepsy due to the pleiotropic manifestations of mutated genes. Based on these new sequencing technologies, exome sequencing may identify other genes responsible for the glucose transport across the blood–brain barrier, which may help to overcome this difficulty and suggest novel drug targets [14,28,44].

The molecular genetic testing used in Glut1-DS include sequence analysis were reported in two studies to be 89% and 81% from total probands in the SCL2A1 pathogenic variant. Moreover, in (multi)exon or whole-gene deletions this varies with 11% and 14% and to increase this percentage the variety of methods that may be used are quantitative PCR, long-range PCR, multiplex ligation-dependent probe amplification (MLPA), and chromosomal microarray (CMA) that includes this gene/chromosome segment.

When the patients do not have a pathogenic variant in the tested gene through sequence analysis, the gene may be either at another locus which cannot be detected or covered by the laboratory’s test including the sequence analysis [38].

There are alternative genetic testing strategies, including the multi-gene panel in case of failure with single-gene molecular genetic testing of SLC2A1 through the sequence and deletion/duplication analysis. Due to this, there was a recently reported study in childhood-onset epilepsy (COE) in which the detection rate is relatively small (2.8%) in patients who were clinically suspected to have GLUT1 DS.

To further enhance the genetic testing potential, the multi genes panel analysis is a promising approach [45]. However, exome sequencing, genome sequencing, and mitochondrial sequencing may also be considered.

Recently, developments in pharmacogenomic and precision medicine approaches are promising in the management of GLUT-1 deficiency [15,17]. A new era of precision medicine will continue to establish larger gene discovery consortia with progression to a whole genome sequencing, which will lead to improvements in patients with unexplained epilepsy in the clinic.

The availability of gene-specific patients to monitor the effects of drugs will be important in influencing therapy decisions and prognosis of the disease [23]. Moreover, it will enable and improve the outcomes in terms of predictions and genetic counseling for family planning and enhance the detection of therapeutic and unwanted effects [28].

## 4. Common Pathogenic Variants in GLUT-1 Deficiency

There are common selected pathogenic variants of SLC2A1 which are responsible for GLUT1 DS. The complete list of pathogenic variants responsible for the GLUT1 DS and respective clinical phenotypes was shown in Table 2 and Figure 1.

The absence of the pathogenic mutation in the SLC2A1 gene does not exclude the diagnosis of the GLUT-1 deficiency. There are case reports that described this phenomenon with sporadic mutation detected by Sanger sequencing with reporting of the novel heterozygous variant c.538T>A, p.Met180Lys in the exon 5 of SLC2A1 gene associated with epilepsy expression, paroxysmal dyskinesias provoked by infection, emotional stress, and fasting which were responding well to the ketogenic diet [46,47].

The SLC2A1 pathogenic mutation of the gene in heterozygosity (c.823G>A, p.Ala275Thr), detection should be suspected for the individualizing the KD treatment in absence seizures mainly in the early childhood associated with irregular ictal EEG discharges, mild mental retardation, migraine, microcephaly, drug resistance, and worsening during fasting [48].

Moreover, the SLC2A1 gene mutations studied in children have shown the presence of de novo and heritable paternal origin mutations including the point mutations c.388g>a, p.Gly132Ser, c.634T>T, p.Arg212Cys, and amino acid insertion c.(688-11G>A), Ser_Val227insValProPro [48].

Recently, a new identification method using whole-exome sequencing was developed, which included detection of rare homozygous missense variants (c.526C>T (p.Arg176Trp) and c.629C>T (p.Ala210Val)) in SLC45A1, encoding another cerebral glucose transporter which indicates that recessive mutation in SLC45A1 (second cerebral glucose transporter in addition to GLUT1) which cause intellectual disability and epilepsy. There was a lack of other clinical features associated with GLUT-1 and also hypoglycorrhachia was not documented in individuals tested in this cohort.

Regarding the potential treatment target, there is some promising indication for ketogenic diet in individuals with SLC45A1 mutations, however, further investigation is needed [49].

Attempts in continuing the identification of the new pathogenic variant spectrum in GLUT1DS are recently documented with a new variant the gene of SLC2A1, including c.734A>C, which could serve as a potential variant in the exploring the individualizing approaches in such patients [50].

## 5. Summary

GLUT1 deficiency syndrome is a treatable disorder of glucose transport into the brain caused by a variety of mutations in the SLC2A1 gene which are the cause of different neurological disorders including different types of epilepsy. Since the patients continue to experience seizures due to a pharmacoresistance, an early clinical diagnosis associated with specific genetic testing including SLC2A1 defect could predict drug response and improve safety and efficacy of treatment in the different clinical phenotypes.

Taking this into consideration, new pathogenic variants analyses, clinical phenotype investigations, and evidence are still developing in the improvement of this corresponding genetic detection, new therapeutic approaches which will influence the treatment, decisions, and prognosis of the disease.

The initiation of an alternative energy source with the introduction of a ketogenic diet in patients with GLUT1-DS is an important and promising individualizing strategy to prevent unnecessary anticonvulsant therapy trials, which are usually ineffective.

Even though the ketogenic diet is still continuing to be used efficiently, there is growing research interest in the development of a new diet which will further improve the quality of life in the patients.

## Figures and Tables

**Table 1:** Current treatment options and precautions for GLUT1 deficiency related to epilepsy.

Diet Treatment	Antiepileptic Drugs	Drug Combinations to Avoid with KD	Drugs to Avoid Due to GLUT1 Impairment
Ketogenic (gold standard)	Acetazolamide	Valproate	Phenobarbital, Valproate
Modified Atkins	Topiramate	Zonisamide	Tyrosine kinase inhibitors
Medium chain Triglycerides	Zonisamide	Acetazolamide	Caffeine, Ethanol
Low glycemic index treatment	Phenytoin	Topiramate	Diazepam, Narcotics
Triheptanoin (under investigation)	Carbamazepine	-	Tricyclic antidepressants
Alpha lipoic acid (under investigation)	-	-	General anaesthetics, Chloral hydrate
-	-	-	Guanosine triphosphate analogues

**Table 2:** SLC2A1 pathogenic variants and phenotypes for GLUT1 deficiency syndrome 1.

DNA Nucleotide Change	Predicted Protein Change	dbSNP	Phenotypes
c.49G>T	p.Gly17Ter	-	NA
c.1089delG	p.Trp363Terfs	rs587784391	A
c.1296C>A	p.Tyr432Ter	rs75485205	A
c.1347C>A	p.Tyr449Ter	rs80359828	A
c.1366A>T	p.Lys456Ter	rs80359829	A
c.19_28delAAGCTGACGG	p.Lys7Valfs	rs587784393	A
c.272G>A	p.Gly91Asp	rs80359814	A
c.376C>T	p.Arg126Cys	rs80359818	A,C
c.377G>A	p.Arg126His	rs80359816	A,C
c.574_57delAT	p.lle192Hisfs	rs878853161	A
c.724C>T	p.Gln242Ter	rs794729221	A
c.748C>T	p.Gln250Ter	rs587784396	NA
c.823G>A	p.Ala275Thr	rs121909740	A,B,C
c.847C>T	p.Gln283Ter	rs587784397	A
c.907dupG	p.val303Glyfs	rs7960655334	A,B
c.940G>A	p.Gly314Ser	rs121909739	A,B
c.966_967delCG	p.Ser324Alafs	rs886044287	NA
c.980_981delTG	p.Val327Glyfs	rs80359838	A,B
c.997C>T	p.Arg333Trp	rs80359825	A,C
c.1198C>T	p.Arg400Cys	rs796053263	NA
c.1402C>T	p.Arg468Trp	rs267607059	A,B
c.377G>T	p.Arg126Leu	rs80359816	A,B
c.766_767delAAinsGT	p.Lys256Val	rs80359822	A
c.971C>T	p.Ser324Leu	rs796053253	B,C
c.988C>T	p.Arg330Ter	rs80359826	A,B,C
c.277C>T	p.Arg93Trp	rs267607061	C
c.458G>A	p.Arg153His	rs149585781	C
c.464C>T	p.Ala155Val	rs35313240	B
c.634C>T	p.Arg212Cys	rs61750200	A,C
c.667C>T	p.Arg223Trp	rs757188030	B,C
c.844C>T	p.Gln282	rs1057521066	A
c.884C>T	p.Thr295Met	rs80359823	A,C
c.998G>A	p.Arg333Gln	rs111033808	B
c.1372C>T	p.Arg458Trp	rs13306758	A,B
c.400G>A	p.Gly134Ser	rs1057518953	A,C
c.100A>G	p.Asn34Asp	rs587784390	A

Phenotypes: A = early-onset classical phenotype; B = late-onset classical; C = non-classical phenotype; NA = data not available.
